# MXenes for Defense-Oriented Multifunctional Systems: From Synthesis and Property Regulation to Deployment Challenges

**DOI:** 10.3390/ma19132799

**Published:** 2026-07-01

**Authors:** Kunqi Zhang, Tao Su, Jia Long, Yipeng Cui, Yan Zhou, Zhifang Liu, Caofeng Pan

**Affiliations:** 1 Institute of Atomic Manufacturing, International Institute for Interdisciplinary and Frontiers, Beihang University, Beijing 100191, China; 2 Beijing Key Laboratory for Atomic Manufacturing Equipment and Intelligent Sensing, Beijing 100191, China

**Keywords:** MXenes, multifunctional defense materials, surface and interface engineering

## Abstract

MXenes, a rapidly expanding family of two-dimensional transition-metal carbides and nitrides, are increasingly viewed as strong candidates for defense-oriented multifunctional systems because they combine metallic conductivity, surface tunability, mechanical flexibility, and solution processability within a lightweight platform. Unlike conventional metals, ceramics, and semiconductors, which usually optimize one or two parameters at the expense of density, brittleness, or integration compatibility, MXenes offer a rare opportunity to coordinate electromagnetic, mechanical, thermal, and sensing functions within one material family. Different from existing reviews that focus on laboratory-level record performance or single-function optimization, this review presents an innovative deployment-oriented perspective and fills the research gap of systematic military-oriented evaluation for MXenes. In this review, we examine MXenes from a deployment-oriented perspective rather than through isolated record values. We first summarize their formation chemistry and major synthesis routes, including HF and in-situ HF etching, bifluoride and alkaline methods, molten-salt strategies, electrochemical approaches, and precursor-free chemical vapor deposition. We then discuss the principal levers of property regulation, focusing on composition design, surface-termination control, and heterostructure engineering, and show how these strategies shape the performance envelopes relevant to shielding, stealth, impact response, energy storage, and sensing. This review constructs a full-chain analytical framework from synthesis, property regulation to military application and deployment challenges for the first time. Finally, we identify the main barriers to translation, especially manufacturing inconsistency, termination heterogeneity, oxidation and interfacial degradation, and limited application-level validation, and outline the most realistic paths toward deployable defense technologies.

## 1. Introduction

In modern defense systems, superiority is no longer defined only by platform speed, structural strength, or nominal firepower. It is increasingly determined by whether a system can regulate electromagnetic exposure, suppress thermal signatures, maintain information integrity, and survive under coupled mechanical and environmental stress [1,2]. Aircraft, unmanned platforms, wearable soldier systems, intelligent armor, and distributed sensor networks must operate under multiband surveillance, repeated deformation, thermal shock, moisture, corrosion, and electromagnetic interference [3]. In this setting, a useful material can no longer be judged by a single optimized metric. It must balance attenuation capability, low weight, flexibility, environmental durability, and, increasingly, the capacity to sense or adapt during service [4,5,6,7,8,9,10,11,12,13].

As the core of military electronic and sensing systems, semiconductor materials provide key support for information perception, transmission and processing. Typical defense-oriented semiconductor materials include gallium nitride (GaN), silicon carbide (SiC), indium gallium arsenide (InGaAs), mercury cadmium telluride (HgCdTe), zinc oxide (ZnO) and diamond. GaN and SiC are widely used in high-power radar and high-temperature electronic devices owing to their wide bandgap and high stability; HgCdTe is the dominant material for infrared detection and thermal imaging reconnaissance; InGaAs shows excellent photoelectric response in near-infrared communication and target recognition; ZnO and diamond have unique advantages in extreme environment sensing and high-performance signal conversion. However, conventional metals, ceramics and the above semiconductors rarely satisfy the coordinated demands of defense systems, since the improvement of single performance is often at the cost of density, brittleness, interfacial mismatch or integration difficulty. Notably, two-dimensional materials including MXene are widely and practically employed as high-efficiency lubricant additives to minimize friction and wear in mechanical systems, thanks to their characteristic layered structure and favorable dispersibility in water-based and engineered lubricants [14,15].

Since 2011, MXene, as a rapidly developing family of two-dimensional transition-metal carbides and nitrides, have shown outstanding structural and functional advantages [16,17,18,19,20]. MXene combine metallic conductivity, flexible layered structure, tunable surface terminations and solution processability, which are distinct from traditional two-dimensional materials [21,22,23,24,25,26]. Such unique characteristics enable MXene to simultaneously realize electromagnetic attenuation, strain response, photothermal regulation and electrochemical energy storage, showing irreplaceable potential in electromagnetic shielding, adaptive camouflage, intelligent protection and extreme-environment energy supply [19,27,28,29,30,31,32,33,34,35].

The rapid development of MXene has attracted extensive academic attention, but most studies focus on laboratory performance optimization rather than deployment-oriented practical requirements. The performance of MXene is highly sensitive to synthesis conditions, surface terminations and structural states, which directly determine the conductivity, stability and reproducibility in practical scenarios [36,37,38]. More importantly, defense systems face coupled constraints of lightweight, broadband functionality, environmental durability and scalable production, which are quite different from the evaluation indicators in basic research. Materials with excellent single performance in laboratories often fail to meet the long-term service requirements under humidity, thermal cycling, salt corrosion and repeated deformation.

Although MXene research has expanded rapidly, most existing reviews still emphasize synthesis chemistry, broad functional applications, or general roles in energy and environmental systems. MXene have been less frequently evaluated from the standpoint of defense-oriented applications, where synthesis methods, property modulation, and deployment contexts are closely interconnected and should not be treated as isolated issues. The practical standards for defense systems differ significantly from academic laboratory metrics, and reviews focused on record-level performance tend to overlook the key obstacles that restrict real-world military implementation.

This review first summarizes the formation mechanism and mainstream preparation methods of MXene. Then, the property regulation strategies including composition design, surface termination control and heterostructure engineering are systematically discussed. On this basis, the applications of MXene in electromagnetic shielding, intelligent protection, extreme-environment energy storage and sensing transmission are comprehensively evaluated. Finally, the key bottlenecks restricting the military deployment of MXene are summarized, and the future development direction is prospected [39].

## 2. Origin and Structural Basis of MXene

**Figure 1:**
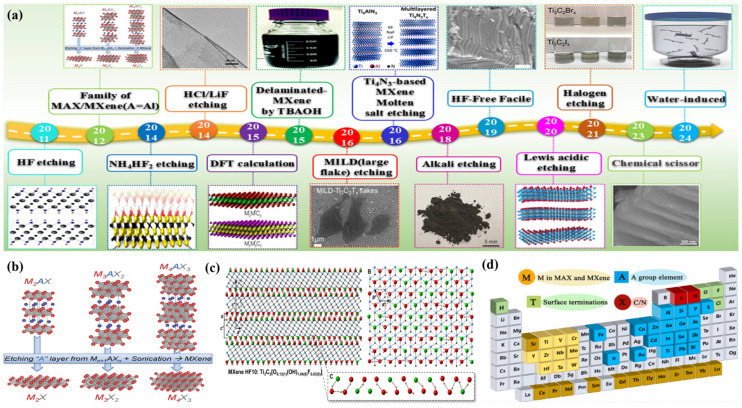
Origin, structural fundamentals and compositional diversity of MXene. (**a**) Timeline summarizing key milestones in the development of MXene synthesis and functionalization from 2011 to 2024, including representative etching strategies and delamination methods [19]. Reprinted with permission from [19]. Copyright 2025 Wiley-VCH GmbH. (**b**) Schematic illustration of the transformation from MAX phases (general formula M*_n_*_+1_AX*_n_*, n = 1–3) to MXene via selective etching of the A-layer and subsequent exfoliation into 2D nanosheets [16]. Reprinted with permission from [16]. Copyright 2013 Wiley-VCH GmbH. (**c**) Atomic-scale structural model of Ti_3_C_2_T_x_ MXene, depicting the layered transition-metal carbide framework and typical surface terminations (-O, -OH, -F) [18]. Reprinted with permission from [18]. 2016 American Chemical Society. (**d**) Compositional diversity of the MXene family mapped on the periodic table, showing the available elements for the M (transition metals), A (group elements), X (C/N), and T (surface terminations) sites [19]. Reprinted with permission from [19]. 2025 Wiley-VCH GmbH.

MXenes originate from a class of layered ternary compounds known as MAX phases, whose general formula is M*_n_*_+1_AX*_n_* (n = 1–3) [40]. As illustrated in Figure 1b, these materials consist of alternating M–X slabs and A-element layers, where strong covalent–ionic bonding within the M–X framework coexists with relatively weaker metallic bonding between M and A layers. This intrinsic bonding asymmetry provides the structural basis for the transformation from MAX phases to MXene, enabling the selective removal of A layers while largely preserving the integrity of the transition-metal carbide or nitride framework. The first successful realization of this concept, achieved through the etching of Ti_3_AlC_2_ in 2011, marked the emergence of MXene as a new family of two-dimensional materials [41,42,43,44]. The key developmental milestones of MXene from 2011 to 2024 are systematically summarized in Figure 1a.

Following A-layer removal, the resulting MXene retain a layered morphology but undergo significant chemical and structural reconfiguration. As shown in Figure 1c, the exposed transition-metal surfaces become terminated by functional groups such as -O, -OH, and -F, which are introduced during the etching process. They play a central role in determining the material’s electronic structure, interfacial behavior and environmental stability. At the same time, the stacked configuration inherited from MAX phases is partially preserved, leading to multilayered MXene with tunable interlayer spacing. The presence of intercalated ions or molecules further expands the interlayer distance, facilitating subsequent delamination into few-layer or single-layer nanosheets.

The compositional and structural diversity of MXene is another defining characteristic. As summarized in Figure 1d, different combinations of transition metals and X elements give rise to a broad family of materials with distinct electronic and chemical properties. This diversity, combined with the variability of surface terminations, creates a highly flexible platform for tailoring material behavior. However, it also introduces significant complexity, as variations in composition and surface chemistry can lead to substantial differences in conductivity, stability, and processability.

These structural and chemical features collectively give rise to the multifunctional properties of MXene. MXene exhibit high electrical conductivity, mechanical flexibility, and strong interaction with electromagnetic waves, enabling applications in electromagnetic shielding, sensing, and thermal management. Importantly, these properties are not independent but are closely linked to the material’s layered structure, surface terminations, and interlayer interactions. Consequently, MXene should be understood not as a single material with fixed characteristics, but as a structure- and process-dependent system.

It should also be noted that the properties described above are highly sensitive to synthesis conditions. Differences in etching chemistry, precursor composition, and post-treatment processes can significantly alter surface terminations, defect density, and interlayer structure, thereby affecting the overall performance of MXene. Therefore, a systematic understanding of MXene synthesis is essential for controlling their properties and enabling their practical application. In the following section, the mechanisms underlying MXene formation and the main preparation strategies are discussed in detail.

## 3. Preparation Methods

### 3.1. Mechanisms Underlying the Formation of MXenes

MXenes are produced through the selective extraction of A-site atoms from layered MAX phases (M*_n_*_+1_AX*_n_*, n = 1–3), and the feasibility of this transformation is fundamentally determined by the bonding hierarchy within the precursor [45,46,47,48]. In MAX phases, robust M-X slabs are interleaved with comparatively weak M-A layers. This contrast in bond strength provides the thermodynamic basis for selective etching and also explains why the transformation can preserve a two-dimensional carbide or nitride framework rather than collapse into a disordered product. A mechanistic understanding of how these bonds respond to chemical or electrochemical attack is therefore essential for rational MXene synthesis.

The distinctive properties of MXenes arise not only from removal of the A layer, but also from the terminated layered structures that emerge during and after etching. MAX phases are ternary layered ceramics with the general formula M*_n_*_+1_AX*_n_* (n = 1, 2, or 3), where M is an early transition metal, A is a group IIIA or IVA element, and X is carbon and/or nitrogen. In these structures, M-X layers are linked by strong covalent and ionic interactions, whereas M-A coupling is comparatively metallic and weaker. Once the A layer is extracted, MXenes (M*_n_*_+1_X*_n_*T*_x_*) are formed with surface terminations such as -O, -OH, and -F, which strongly influence electronic structure, wettability, chemical reactivity, and interlayer interactions. Taking Ti_3_C_2_T*_x_* as a typical example, hydroxyl-rich surfaces generally improve compatibility with polar solvents through hydrogen bonding, whereas fluorine-containing terminations can alter stability and transport behavior. During etching, reactive species such as HF, bifluoride-derived fluoride, or halide ions preferentially attack the A-site atoms, forming stable complexes or redox products that weaken and finally break the M-A bonds while largely preserving the robust M-X framework. Removal of A atoms exposes coordinatively unsaturated metal sites, which rapidly react with the surrounding medium to generate terminations including -O, -OH, -F, -Cl, or -Br. At the same time, electrostatic repulsion between adjacent layers increases, and concurrent intercalation of small cations such as Li^+^, Na^+^, or NH_4_^+^ expands the interlayer spacing and produces the characteristic accordion-like morphology [49,50,51,52,53,54]. Subsequent delamination by ultrasonication, mechanical agitation, or solvent exchange can then yield few-layer or monolayer MXene nanosheets [55,56,57].

Taken together, selective disruption of M-A bonds, generation of surface terminations, and progressive interlayer evolution define the mechanistic core of MXene formation. These processes determine more than etching success or delamination yield; they also preconfigure the defect landscape, termination distribution, interfacial reactivity, and oxidation susceptibility that later govern device performance. For deployment-oriented applications, a synthesis method should therefore be evaluated not only by whether it removes the A layer efficiently, but by how reproducibly it controls the chemical and structural states that remain after etching.

### 3.2. Mainstream Preparation Technologies

Over the past decade, MXene synthesis has evolved from a small set of strong-acid protocols into a much broader toolkit of milder, more controllable, and in some cases precursor-free strategies. This shift reflects three persistent demands: safer processing, finer control over defects and surface terminations, and greater compatibility with scalable manufacturing. For defense-oriented technologies, these issues are not secondary engineering details. They directly determine whether a material can move beyond promising laboratory data toward reproducible performance under realistic service conditions. A summary of representative MXene synthesis methods, including their mechanisms, advantages, and limitations, is provided in Table 1.

#### 3.2.1. Wet Chemical Etching

**Figure 2:**
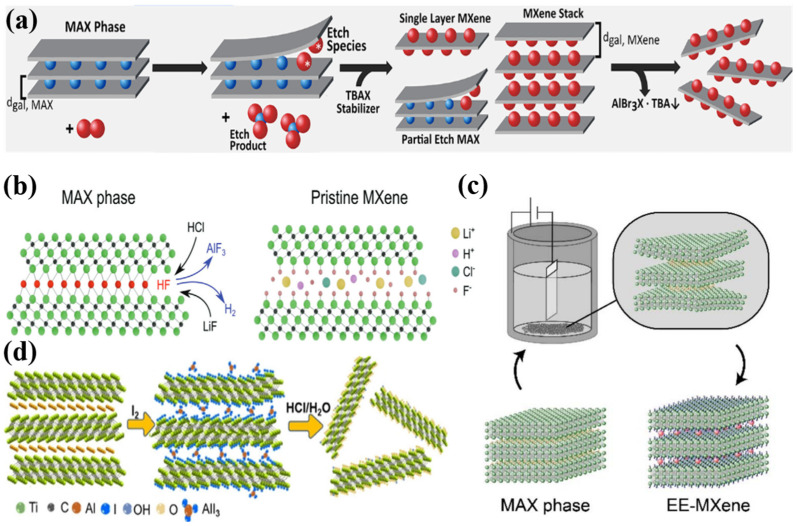
Synthesis pathways and representative mechanisms for MXene fabrication. (**a**) Schematic illustration of the general etching-delamination process, in which the A layer in the MAX phase is selectively removed, followed by stabilization and intercalation to yield single-layer MXene and stacked assemblies [74]. Reprinted with permission from [74]. Copyright 2021 American Chemical Society. (**b**) Atomic-scale mechanism of LiF/HCl-assisted etching, showing in situ HF generation, A-layer extraction, surface termination formation (-O, -OH, -F), and evolution from the MAX phase to pristine MXene structures [60]. Reprinted with permission from [60]. Copyright 2025 Wiley-VCH GmbH. (**c**) Schematic representation of electrochemical etching-exfoliation synthesis [75]. Reprinted with permission from [75]. Copyright 2024 American Chemical Society. (**d**) Halogen-assisted etching and post-treatment route [76]. Reprinted with permission from [76]. Copyright 2021 Wiley-VCH GmbH.

Wet chemical etching remains the most established route for MXene preparation and continues to define the baseline against which newer methods are judged. These approaches use liquid-phase etchants to selectively dissolve the A-site layers of MAX precursors while largely preserving the transition-metal carbide or nitride framework. Their importance lies not only in synthetic simplicity, but also in the broad access they provide to flake production, termination engineering, and post-etch intercalation. At the same time, the maturity of wet etching should not be mistaken for full controllability, because differences in acid chemistry, intercalants, and post-treatment can generate markedly different defect populations and termination states. The general etching-delamination sequence is schematically shown in Figure 2a.

Hydrofluoric acid (HF) etching. The classical HF route marked the starting point of modern MXene chemistry. Its main advantages are speed, relatively complete A-layer removal, and access to large lateral flakes [58]. MXene prepared by HF etching exhibits high crystallinity but carries abundant fluorine-containing terminations, which reduce surface hydrophilicity and interfacial compatibility, and slightly suppress intrinsic electrical conductivity. Yet from a deployment standpoint, its drawbacks are equally fundamental. Severe corrosiveness and toxicity hinder scale-up, while the strong fluorinating environment often leaves products rich in fluorine-containing terminations. Such surfaces are acceptable for many proof-of-concept studies, but they may be suboptimal when high hydrophilicity, electrochemical accessibility, or precise interfacial control is required. HF etching therefore remains historically foundational and experimentally effective, but it is increasingly viewed as a synthesis benchmark rather than an ideal endpoint for application-driven manufacturing [27].

In situ HF etching (LiF/HCl system). Generating HF in situ from fluoride salts and acid was a major advance because it improved handling safety while simultaneously introducing intercalant species that facilitate delamination [59,60,61]. The as-prepared MXene possesses abundant oxygen- and hydroxyl-rich terminations, significantly enhancing electrical conductivity, dispersibility in polar solvents, and electrochemical activity compared with HF-etched counterparts; meanwhile, Li^+^ intercalation enlarges interlayer spacing, endowing the product with superior exfoliation yield and flexible film-forming ability. The LiF/HCl system is especially important because lithium ions expand the galleries during etching, often producing materials that are easier to exfoliate and richer in oxygen- and hydroxyl-containing terminations. The mechanistic details of LiF/HCl-assisted etching are illustrated in Figure 2b. The broader significance of this route is that it begins to couple etching chemistry with structural control rather than treating them as separate steps. Recent in situ X-ray diffraction (XRD) studies have provided real-time dynamic insights into the structural evolution during etching, clearly revealing the gradual disappearance of MAX phase characteristic diffraction peaks and the simultaneous emergence of MXene diffraction signals. These studies quantitatively tracked the continuous expansion of interlayer spacing from ~1.9 nm (pristine MAX phase) to more than 2.5 nm upon Li^+^ intercalation, and identified three sequential kinetic stages (induction period, rapid etching stage, and steady-state stage) that dominate the MAX-to-MXene conversion process. Such real-time structural monitoring has further clarified how interlayer spacing evolves during processing, moving this method from empirical optimization toward mechanism-guided synthesis [60]. Even so, the route still leaves substantial batch-to-batch variability, and its practical value depends on whether that variability can be narrowed enough for reproducible device fabrication.

Bifluoride etching. Bifluoride-based systems provide a useful compromise between strong-acid etching and more aggressively engineered routes. By combining moderated fluoride release with cation intercalation, they can simplify subsequent exfoliation and produce enlarged interlayer spacing [62,63]. MXene synthesized via bifluoride etching exhibits larger interlayer distance and better thermal stability than HF-etched samples, with more balanced electrical conductivity and mechanical flexibility. In some reports, bifluoride-derived MXene also exhibit improved thermal stability relative to conventionally HF-etched materials. Their main attraction lies in process gentleness and structural expansion, but this comes with a different challenge: the resulting termination landscape is often more complex and harder to standardize. For applications that are highly sensitive to interfacial chemistry, that complexity may offset the apparent processing advantage.

Alkaline etching. Fluorine-free alkaline routes represent an important conceptual step because they decouple MXene formation from fluoride-rich chemistries and can favor oxygen- and hydroxyl-terminated surfaces [64,65]. The fluorine-free feature eliminates surface fluorine defects, yielding MXene with superior hydrophilicity, electrochemical activity, and biocompatibility; such products are more suitable for flexible devices, sensing, and energy storage compared with fluoride-containing etched MXene. This is attractive when electrochemical accessibility, interfacial compatibility, or environmental concerns make fluorine-containing terminations undesirable. At the same time, alkaline synthesis typically relies on concentrated base under elevated temperature and pressure, which introduces its own barriers to continuous manufacturing. In other words, alkaline etching improves one set of constraints while tightening another, illustrating a recurring theme in MXene synthesis: no route is universally superior, and each must be judged against the requirements of the intended application.

#### 3.2.2. High-Temperature Halide-Driven Etching

High-temperature molten-salt etching broadens both the compositional family of MXenes and the range of accessible surface terminations. It is particularly valuable for MAX phases that are difficult to convert by conventional wet-chemical methods, and it has become a key route for accessing unusual chemistries that are relevant to high-temperature or application-specific environments. Compared with wet chemical etching, molten-salt methods offer stronger etching capability, higher crystallinity retention, and wider compositional adaptability, especially for nitride and high-melting-point MXene that cannot be prepared by low-temperature etching.

Fluorinated molten-salt etching. Early demonstrations of molten-salt etching enabled the synthesis of nitride MXenes, for example by converting Ti_4_AlN_3_ in fluoride-based eutectic salts [66,67]. This method achieves highly crystalline MXene with complete structural integrity, low defect density, and excellent high-temperature stability; nitride MXene prepared hereby exhibits superior electrical conductivity and oxidation resistance compared with carbide MXene from wet methods. The high-temperature ionic environment provides strong etching capability and is especially useful for nitride MAX phases that are difficult to process in aqueous systems. The resulting products often exhibit high crystallinity, although they also tend to remain tightly stacked and are therefore more difficult to delaminate. More recently, fluorinated molten salts have been used to prepare fluorine-terminated MXene nanosheets that can be stably dispersed after appropriate post-treatment. The main value of this route lies in expanding synthetic accessibility rather than in offering the simplest pathway to monolayer delamination.

Lewis acid molten-salt etching. In 2019, Li et al. introduced Lewis acidic molten salts such as ZnCl_2_ as etchants for MAX phases, opening a redox-driven extraction route rather than a purely fluorination-based one [68]. This method realizes precise control over halogen terminations (-Cl, -Br), endowing MXene with tunable work function, charge transport behavior, and interfacial bonding strength; the one-step formation of metal-decorated MXene further enhances electromagnetic attenuation and catalytic performance. This strategy removes the A-site element while simultaneously introducing halogen terminations such as -Cl or -Br. Chloride-terminated MXenes, for example, can be synthesized by etching suitable MAX precursors in ZnCl_2_ melts. The significance of this method extends beyond A-layer removal: it provides a route to deliberate atomic-level control over surface chemistry and, in some systems, enables one-step preparation of MXenes decorated with metal species supplied by the molten salt itself [68,69,70]. A representative halogen-assisted etching and post-treatment route for termination engineering is schematically shown in Figure 2d.

The emergence of Lewis acid molten-salt etching marked an important shift in MXene synthesis, from simply producing exfoliable sheets to deliberately engineering surface atomic structure. Halogen-terminated MXene can act as versatile intermediates because -I, -Cl or -Br groups may subsequently be replaced by non-halogen terminations such as -O, -S, or -NH through post-synthetic treatment. This degree of surface control is particularly valuable when conductivity, catalytic behavior, infrared emissivity, or interfacial bonding must be tuned in a coordinated manner. The trade-off is that these routes often yield multilayer products and require more demanding thermal processing.

#### 3.2.3. Other Methods

Electrochemical etching. Electrochemical synthesis offers an environmentally friendlier and potentially more controllable route to MXenes. In 2017, selective electrochemical removal of the A layer from Ti_2_AlC in dilute HCl was reported as an HF-free strategy for MXene preparation [69,70,71]. This green route produces MXene with uniform oxygen/hydroxyl terminations, low defect concentration, and stable electrical conductivity; the smooth surface and clean interface improve the structural stability and durability of composite films, which is highly competitive for flexible electronics and military wearable devices. The electrochemical etching-exfoliation process is summarized in Figure 2c. In this approach, the MAX phase serves as the working electrode, and an applied potential drives selective oxidation and dissolution of the A-site element. By adjusting electrolyte composition and voltage window, it is possible to influence the surface terminations of the resulting product, typically enriching -O, -OH, and related groups. Early electrochemical methods were limited by low yield, shallow etching depth, and over-etching that could generate carbide-derived carbon. More recently, pulsed-current electrochemical etching has helped alleviate these issues by producing transient hydrogen bubbles that refresh the interface, mitigate passivation, and prolong reaction activity. This progress makes electrochemical routes increasingly attractive for sustainable and scalable production.

Chemical vapor deposition (CVD). CVD represents a bottom-up, MAX-precursor-free route in which MXene layers are grown directly from gaseous transition-metal and carbon or nitrogen precursors on heated substrates [72,73]. CVD-grown MXene films feature atomic-level flatness, ultra-high purity, and excellent in-plane electrical conductivity, far exceeding the performance of etched MXene in microelectronic devices; the precise thickness control also enables customized electromagnetic response for stealth applications. The main attraction of this method is atomic-level control over film thickness, crystallinity, and termination chemistry, making it especially appealing for wafer-scale high-quality films and microelectronic integration. It also opens access to compounds that are difficult to obtain through conventional MAX-phase etching. However, the high cost, demanding process control, and relatively low throughput of CVD currently limit its use primarily to fundamental studies and specialized thin-film fabrication rather than bulk powder production for structural or composite applications.

## 4. Regulation Methods and Properties of MXenes

Following synthesis and post-treatment, MXenes could display a wide range of attractive properties. For practical deployment, however, it is not enough to catalogue those properties one by one. The more important task is to identify which structural variables control them, what trade-offs emerge when one property is optimized over another, and how reproducibly those relationships can be maintained across batches and architectures. This section therefore focuses not on isolated performance claims, but on the regulation strategies that govern MXene behavior and on the core properties that most directly determine practical value in advanced military and engineering contexts.

### 4.1. Regulation Methods

#### 4.1.1. Composition Regulation

Composition design is the first major lever for tuning MXene behavior because the choice of transition-metal species and carbide or nitride backbone determines electronic structure, bonding character, oxidation susceptibility, and mechanical stiffness at the most fundamental level [77,78]. The issue is not simply that different compositions give different properties; rather, they occupy different positions within a trade space defined by conductivity, redox activity, robustness, and environmental stability [79,80,81,82,83]. Ti-, V-, Mo-, and Nb-based MXenes should therefore not be treated as interchangeable members of one generic material class. Mixed-metal MXene further enlarge this space by enabling conductivity, stiffness, and chemical stability to be balanced within one framework. From a deployment perspective, composition is thus not a secondary optimization variable but a first-order design choice that determines which application windows are realistically accessible.

#### 4.1.2. Surface-Termination Regulation

**Figure 3:**
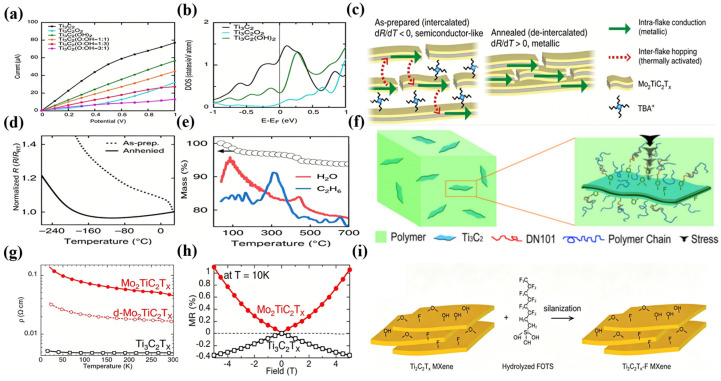
Multifaceted properties of MXene. (**a**) Electrical transport characteristics of MXene with different surface terminations, showing termination-dependent current-voltage behavior and conductivity modulation [100]. (**b**) Electronic structures of Ti_3_C_2_-based MXene, where variations in surface terminations lead to distinct densities of states near the Fermi level [100]. Reprinted with permission from [100]. Copyright 2023 AIP Publishing. (**c**) Charge-transport mechanisms in MXene, illustrating the transition from interflake hopping in intercalated states to intraflake metallic conduction after de-intercalation or annealing [101]. (**d**) Temperature-dependent resistance behavior of MXene, reflecting changes in transport regimes and carrier scattering mechanisms [101]. (**e**) Thermal stability and surface chemistry evolution of MXene under heating, as revealed by mass-loss and atmosphere-dependent analyses [101]. Reprinted with permission from [101]. Copyright 2019 Springer Nature. (**f**) Schematic illustration of MXene-polymer composites under mechanical stress, highlighting interfacial interactions and conductive pathway evolution relevant to strain sensing [102]. Reprinted with permission from [102]. Copyright 2016 Springer Nature. (**g**) Temperature-dependent resistivity of different MXene, demonstrating the influence of composition and defects on electrical transport [103]. (**h**) Magnetoresistance responses of MXene at low temperatures, revealing distinct electronic behaviors under applied magnetic fields [103]. Reprinted with permission from [103]. Copyright 2018 Springer Nature. (**i**) Surface functionalization of MXene via chemical modification, illustrating routes to tailor surface chemistry and interfacial compatibility [104]. Reprinted with permission from [104]. Copyright 2024 Elsevier.

Surface terminations sit at the center of MXene property control because they directly influence charge distribution, wettability, interlayer coupling, dispersibility, work function, and chemical reactivity. As shown in Figure 3a,b, electrical transport and electronic structures vary significantly with termination chemistry. Varying the relative abundance of groups such as -O, -OH, and -F can therefore reshape transport behavior, environmental stability, and stimulus response in ways that are often as important as the underlying carbide or nitride lattice itself [84,85,86,87]. Hydroxyl-rich surfaces usually improve polarity and compatibility with polar media, which can be advantageous for dispersion, sensing, and electrochemical access, whereas excessive fluorine termination may hinder matrix compatibility and complicate interfacial engineering. Representative routes to tailor surface chemistry are shown in Figure 3i. The practical implication is that termination control links synthesis directly to use: if terminations remain poorly defined or unstable, device-level behavior will remain difficult to reproduce regardless of how attractive the intrinsic conductivity appears.

#### 4.1.3. Heterostructure Engineering

Heterostructure engineering provides a practical route for compensating the intrinsic weaknesses of single-component MXenes and for creating multifunctional responses that no isolated MXene film can deliver. By coupling MXenes with ceramics, elastomers, carbon materials, phase-change media, or shape-memory matrices, researchers can redistribute stress, modify charge-transfer pathways, suppress oxidation, and introduce complementary mechanical or thermal functions [88,89,90]. This strategy is especially important in deployment-oriented systems, where performance is constrained by conflicting requirements rather than by one target metric. In electromagnetic and infrared dual-stealth designs, for example, heterointerfaces may be essential for balancing shielding effectiveness, heat transport, emissivity, and durability. Heterostructures should therefore be viewed not as optional embellishments, but as one of the main routes by which MXene-based concepts become application-relevant.

### 4.2. Core Properties

After targeted regulation and structural optimization, MXenes exhibit a set of core properties that explain their broad appeal. Yet for deployment-oriented analysis, these properties must be discussed in terms of their structural origins, their practical limits, and the compromises they impose in real systems. The following discussion therefore emphasizes not only what MXenes can do, but also why performance varies so strongly and where apparently attractive laboratory metrics can become fragile under realistic service conditions.

To move beyond descriptive comparison, it is useful to introduce a deployment-oriented evaluation framework based on three quantitative criteria: (i) absolute performance (e.g., conductivity, shielding effectiveness), (ii) performance retention under environmental stress (humidity, oxidation, thermal cycling), and (iii) system-level efficiency such as performance per unit thickness or weight. Within this framework, MXene should not be judged solely by peak laboratory values, but by their position relative to competing materials such as graphene, carbon nanotubes, and conventional metals under realistic operating conditions. This perspective helps distinguish between record-level performance and practically sustainable functionality.

Electrical properties. MXene exhibit electrical conductivities comparable to or exceeding many conventional EMI-shielding materials. MXene are widely valued for their high room-temperature conductivity, which arises from the metallic or near-metallic character of their transition-metal carbide or nitride frameworks and is further modulated by interlayer spacing, defect density, and termination chemistry [91,92,93,94,95]. In well-processed systems, conductivity can reach the order of 10^5^–10^6^ S/m, but this should not be interpreted as a fixed intrinsic number. For comparison, this conductivity range is comparable to reduced graphene films (10^4^–10^5^ S/m) and lower than bulk metals such as copper (~10^7^ S/m), but MXene often outperform these materials in thickness-normalized shielding efficiency due to their layered morphology and internal multiple scattering mechanisms. However, such advantages are highly condition-dependent. Under ambient exposure, oxidation can reduce conductivity by more than one order of magnitude within days, especially for single- or few-layer MXene. Moisture adsorption further modifies interflake resistance by altering interlayer spacing and contact quality. As a result, the effective conductivity in practical devices is often governed by environmental stability rather than intrinsic electronic structure. Carrier transport depends strongly on interflake contact resistance, residual intercalants, stacking disorder, and termination-induced scattering. The transition between interflake hopping and intraflake metallic conduction is depicted in Figure 3c. As a result, electrical performance in devices often reflects microstructural quality as much as lattice chemistry. MXene also display useful electrochromic behavior because ion intercalation and charge-density modulation can reversibly change optical transmittance. Gogotsi’s group (Drexel University) has realized mature applications of such properties in high-performance EMI shielding films and flexible transparent electrodes, which represent the most typical practical utilization of MXene conductivity. From a defense perspective, the key point is that electrical tunability is valuable only when it remains stable under environmental and mechanical disturbance; otherwise, high initial conductivity can provide a misleading picture of practical readiness. Temperature-dependent resistance behavior of MXene is presented in Figure 3d. Figure 3g,h compares temperature-dependent resistivity and magnetoresistance across different MXenes.

Mechanical properties. MXenes can combine high in-plane stiffness with substantial tensile strength because strong metal-carbon or metal-nitrogen bonding is coupled with layered architectures that still allow limited intersheet sliding [96,97,98]. Typical in-plane elastic modulus of Ti_3_C_2_T*_x_* MXene reaches ~300–500 GPa, tensile strength up to 200–350 MPa, and fracture strain ranges from 0.15% to 0.3% for single-layer nanosheets; for MXene-polymer composites, the tensile strength can be tailored between 15–80 MPa with elongation at break improved to 8–20% to meet flexible military device requirements. Their mechanical response, however, is highly architecture-dependent. Cross-linkers and composite matrices can improve load transfer and toughness, while preserving flexibility that would be difficult to achieve in monolithic ceramics or metals. MXenes are also notable for pronounced piezoresistive sensitivity, because deformation reconfigures intersheet contacts, conductive pathways, and tunneling distances. This makes them attractive for structural monitoring and intelligent protection. Sun’s group (Shanghai Institute of Ceramics, CAS) has developed reliable flexible strain sensors via these piezoresistive characteristics, which have been verified in practical wearable device prototypes. The caution is that mechanical sensitivity and mechanical durability are not identical: a highly responsive network may still suffer from fatigue, microcracking, or interfacial degradation during repeated service, especially in layered films and soft composites. Fatigue testing in cyclic deformation conditions has shown that MXene-based conductive networks may suffer from progressive microcrack formation and interfacial debonding, leading to signal drift or permanent conductivity loss after 10^3^–10^5^ cycles depending on composite design. This highlights a key trade-off: structures optimized for high gauge factor often rely on fragile percolation networks, whereas mechanically robust systems tend to sacrifice sensitivity. From a deployment perspective, fatigue-resistant architectures are therefore more critical than peak sensing performance. The underlying mechanism of this piezoresistive response is illustrated in Figure 3f, which shows how mechanical stress reconfigures intersheet contacts and conductive pathways in MXene-polymer composites.

Extreme-environment stability. Unmodified MXenes are generally vulnerable to oxidation and hydrolysis, and this weakness is central rather than peripheral for defense deployment. Materials must tolerate coupled stresses involving heat, humidity, salt, radiation, and repeated mechanical loading without rapid loss of conductivity or structural integrity. In the defense industry, typical structural materials such as Ti-6Al-4V alloy suffer severe performance degradation from salt spray corrosion and electrochemical erosion. Protective coatings, barrier layers, and composite design have significantly improved resistance to high temperature, corrosion, and irradiation, showing that environmental fragility is not an unavoidable limitation. This corrosion risk in military equipment has been validated in relevant anticorrosion studies on Ti-6Al-4V alloy [99]. Zhao group has applied the stabilized MXene in long-life anti-corrosion coatings for metal components, achieving validated practical protection effects. Figure 3e shows the thermal stability and surface-chemistry evolution under heating. Even so, stability remains one of the strongest filters separating laboratory demonstrations from realistic deployment. From a benchmarking perspective, MXene-based films can achieve shielding effectiveness (SE) values above 60–90 dB at thicknesses below 100 μm, which is comparable to or exceeds many graphene- and CNT-based composites at similar thickness. In contrast, conventional metals such as aluminum or copper require significantly greater thickness to achieve similar attenuation, although they retain superior long-term stability. When normalized by density or thickness, MXene often exhibit higher specific shielding efficiency, but this advantage diminishes under oxidation or structural degradation. Therefore, the competitive position of MXene lies not in absolute shielding performance, but in lightweight and multifunctional integration. A material that performs well for hours or days in controlled tests may still be unsuitable for field use if its terminations evolve, interfaces degrade, or layered structures delaminate under long-term coupled exposure.

## 5. Military Applications

### 5.1. Electromagnetic Shielding and Dynamic Stealth

**Figure 4:**
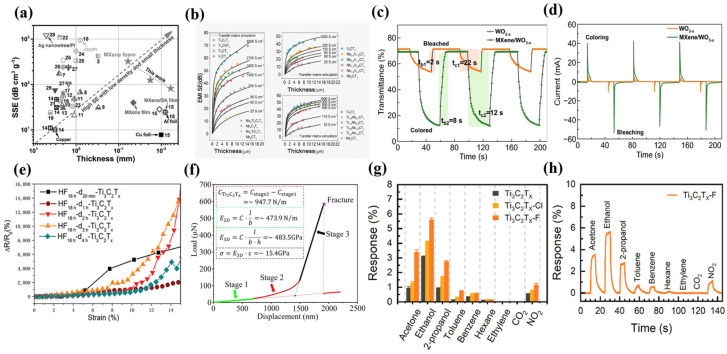
Multifunctional performance of MXene-based materials for military applications. (**a**) Comparison of specific electromagnetic interference (EMI) shielding effectiveness (SSE) versus thickness for MXene materials and conventional/competitive materials, highlighting MXene’s lightweight advantage for compact defense systems [28]. Reprinted with permission from [28]. 2017 Wiley-VCH GmbH. (**b**) EMI shielding effectiveness (SE) of various MXene compositions as a function of thickness, including transfer matrix simulation data [4]. Reprinted with permission from [4]. 2024 Wiley-VCH GmbH. (**c**,**d**) Electrochromic performance of MXene/WO_3-x_ composites: (**c**) Transmittance modulation during bleaching/coloring cycles, showing faster switching times than pure WO_3−x_ for visual impact damage warning; (**d**) Corresponding chronoamperometric current responses [119]. Reprinted with permission from [119]. 2020 Springer Nature. (**e**) Piezoresistive strain response of Ti_3_C_2_T_x_ prepared under different etching conditions, plotted as relative resistance change versus tensile strain, for structural health monitoring [29]. Reprinted with permission from [29]. 2019 Wiley-VCH GmbH. (**f**) Nanoindentation load-displacement curve of MXene, revealing its mechanical deformation behavior and key parameters (elastic modulus, fracture strength) that underpin impact resistance [23]. Reprinted with permission from [23]. 2024 Springer Nature. (**g**,**h**) Gas sensing performance of MXene with different surface terminations: (**g**) Comparative response to volatile organic compounds (VOCs) and gaseous analytes; (**h**) Dynamic response curves of Ti_3_C_2_T_x_-F for battlefield hazard detection [41]. Reprinted with permission from [41]. 2020 American Chemical Society.

MXene-based materials have become some of the most intensively studied candidates for lightweight electromagnetic management because their high conductivity and layered morphology naturally support reflection, absorption, and repeated internal attenuation of incident waves [105,106,107]. Relative to conventional metallic shields, MXene films, foams, and porous composites can deliver strong shielding at lower density and with greater flexibility, which is particularly attractive for aircraft, unmanned systems, and portable equipment. As shown in Figure 4a, MXene-based materials achieve high specific shielding effectiveness at significantly reduced thickness compared with conventional metallic counterparts, highlighting their unique lightweight advantage for compact defense systems. Their appeal for dynamic stealth lies in the possibility of actively modulating electrical and thermal responses rather than relying on static attenuation alone. Even so, the true challenge is not whether MXenes can achieve high shielding effectiveness in thin samples, but whether they can maintain broadband, thickness-efficient, and environmentally stable performance while simultaneously satisfying constraints on infrared signature, mechanical reliability, and scalable manufacturing. As shown in Figure 4b, the shielding effectiveness of various MXene compositions increases monotonically with film thickness, and transfer matrix simulations further validate the thickness-dependent attenuation behavior of layered MXene architectures. In this sense, electromagnetic shielding is one of the clearest examples of MXene promise, but also one of the clearest demonstrations that peak laboratory values do not by themselves establish deployability.

### 5.2. Intelligent Protection and Impact Response

The pronounced piezoresistive response of MXene networks makes them attractive for intelligent protection systems that require both mechanical buffering and electrical self-reporting under impact or shock loading [108,109,110,111]. The electrochromic performance of MXene/WO_3−x_ composites for visualized impact warning is displayed in Figure 4c,d. When these materials are deformed, intersheet contacts and conductive pathways reorganize rapidly, producing measurable resistance changes that can reveal strain localization or damage evolution in real time. This creates opportunities for self-sensing protective structures rather than passive armor alone. In addition, integration with elastomers, ceramics, or metallic hosts can broaden the design space toward impact mitigation, adaptive optical regulation, and structural health monitoring. The mechanical deformation and impact resistance of MXene are revealed by nanoindentation in Figure 4f. The key limitation is that impact sensitivity, ballistic robustness, and long-term fatigue resistance are not automatically aligned. A network optimized for signal output may not be the one best suited to survive repeated high-rate deformation, so intelligent protection must be designed as a coupled structural-electronic problem rather than as a sensor layer added after the fact. The piezoresistive strain response of Ti_3_C_2_T_x_ under different etching conditions is shown in Figure 4e, providing quantitative support for its structural health monitoring capability.

### 5.3. Energy Storage and Monitoring in Extreme Environments

MXene-based supercapacitors and related electrochemical devices are appealing for extreme-environment power systems because layered ion-transport pathways and redox-active surfaces can support fast charge storage, high-rate operation, and useful capacity retention across broad temperature windows [112,113,114]. While gravimetric capacitance values of MXene electrodes can exceed 300–1500 F/g under laboratory conditions, these values are typically measured in controlled electrolytes and short-term cycling. In practical environments, electrolyte evaporation, electrode oxidation, and interlayer restacking can significantly reduce accessible capacitance and rate performance. Compared with activated carbon and graphene-based electrodes, MXene offer faster ion transport but often suffer from lower long-term cycling stability unless protected by structural or chemical stabilization strategies. This highlights the importance of evaluating energy-storage performance not only by peak capacitance but by retention under realistic operating conditions. These features are relevant for mobile platforms, cold-region operations, and thermally harsh service conditions where rapid power delivery and compact form factors are essential. Just as important, the coupling between electrochemical state and electrical response creates opportunities for integrated monitoring, allowing energy devices to function simultaneously as power units and state-diagnostic components. Yet this application area also reveals a critical constraint: electrochemical promise is meaningful only if MXene electrodes retain structural integrity and interfacial stability during long-term cycling, temperature fluctuations, and electrolyte exposure. Otherwise, excellent short-term capacitance data may overstate the readiness of these systems for real battlefield energy management.

### 5.4. Intelligent Sensing and Signal Transmission

MXenes are also highly attractive for intelligent sensing and signal transmission because their surface chemistry supports rapid adsorption-driven electrical responses and their conductive networks can preserve low-impedance pathways in flexible architectures [115,116,117,118]. The gas sensing performance of MXene with different surface terminations is summarized in Figure 4g,h. These characteristics are useful for toxic-gas monitoring, environmental sensing, and signal routing in electromagnetically complex settings. Flexible films and fibers can maintain conductivity during bending and deformation, which is essential for wearable or deployable systems. At the same time, this area is particularly vulnerable to overinterpretation. High sensitivity measured under controlled laboratory conditions does not automatically translate into selectivity, drift resistance, or reliable communication performance in noisy and humid environments. In particular, humidity cross-sensitivity and baseline drift remain major challenges for MXene-based sensors, because surface adsorption processes are not highly selective. Compared with semiconductor-based sensors such as metal oxides, MXenes sensors often exhibit faster response but weaker intrinsic selectivity. Therefore, their practical deployment depends on signal processing, functionalization, or hybridization strategies to suppress interference rather than relying solely on intrinsic material response. For sensing and transmission applications, the decisive issue is therefore not merely responsiveness, but stability, anti-interference capability, and signal integrity under realistic service conditions.

## 6. Future Prospects and Key Challenges

### 6.1. Future Military Application Prospects

Beyond the applications already demonstrated, the most promising future roles of MXenes in military systems are likely to be those that exploit several of their attributes at once rather than pursuing a single record metric. Their real strategic value will probably emerge in architectures where electromagnetic regulation, sensing, structural compliance, and thermal management must be integrated within limited weight and volume budgets. This systems-level perspective is more useful than asking whether MXenes can replace conventional materials outright, because in many cases their strongest contribution will be to reduce functional redundancy and enable lighter, more adaptive multilayer designs.

Individual combat systems. Modern soldier platforms increasingly integrate protection, communication, sensing, thermal regulation, and portable power, and the resulting equipment burden directly affects mobility and mission endurance. MXenes are attractive here because they could serve as multifunctional interlayers that combine shielding, local sensing, mechanical compliance, and possibly thermal or infrared regulation within one lightweight architecture. Their most realistic role is therefore not wholesale substitution of mature materials, but selective integration into wearable sensors, protective textiles, helmet liners, soft armor subsystems, and compact energy modules. The concept is compelling, but it still depends on solving biocompatibility, washability, fatigue durability, and manufacturing consistency at scales relevant to actual gear deployment.

Strategic stealth submarines. Deep-sea platforms operate under coupled conditions of pressure, salinity, limited maintenance access, and extended service lifetimes, placing unusually stringent demands on corrosion resistance, electromagnetic management, thermal regulation, and compact power systems. MXenes are attractive as corrosion-resistant functional interlayers, shielding-related coatings, or compact electrochemical components because their surface and interface chemistry can, in principle, be tuned for several of these tasks simultaneously. However, this scenario should be regarded as a forward-looking systems prospect rather than a near-term application claim. Long-term encapsulation, chemical stabilization, adhesion under hydrostatic stress, and service-life validation remain unresolved, and these factors will determine whether MXenes can move beyond conceptual relevance in such platforms.

When it comes to device preparation, benefiting from their tunable electrical, mechanical and chemical responsiveness, MXenes can be used to construct versatile high-sensitivity sensors, mechanical and chemical responsiveness, MXenes can be used to construct versatile high-sensitivity sensors, including piezoresistive strain/pressure sensors, electrochemical gas sensors, humidity/temperature sensors, and photoelectric/infrared photothermal sensors. In addition, a variety of core functional elements can be fabricated based on MXene, such as flexible conductive electrodes, electromagnetic shielding films, micro energy storage units, anti-corrosion barrier coatings, and electrochromic adaptive films. These sensor devices and functional elements provide key support for the integrated application of MXene in multifunctional systems.

### 6.2. Challenges for Practical Deployment

To move MXenes toward practical military deployment, the field must address a set of coupled bottlenecks that extend well beyond proof-of-concept demonstrations. These bottlenecks are not isolated. Poor synthesis consistency undermines property control; limited validation obscures realistic performance windows; and environmental fragility amplifies failures once materials are integrated into devices or structures. For this reason, the central challenge is not maximizing one benchmark number, but establishing a reproducible pathway from materials design to component-level reliability.

Scalability and consistency. Industrial adoption requires synthesis routes that can deliver MXenes with controlled flake size, thickness distribution, defect density, oxidation state, and termination chemistry across large batches. At present, production variability remains substantial, and that variability propagates directly into fluctuations in conductivity, shielding behavior, mechanical response, and electrochemical performance. Large-area film fabrication adds a second layer of difficulty because restacking, voids, microcracks, and local oxidation can emerge during assembly and drying. Until scalable processing can produce uniform, defect-minimized MXene architectures with predictable properties, many impressive laboratory demonstrations will remain difficult to reproduce at the component level.

Lack of application-level validation. MXene-based materials have shown excellent results in many laboratory studies, but the available validation datasets remain limited compared with those for conventional metals, engineering polymers, or more mature low-dimensional materials such as graphene. What is often missing is not another record value, but statistically meaningful evidence under realistic service protocols. Defense-relevant adoption will require standardized processing windows, reproducible manufacturing demonstrations, and testing under coupled conditions involving humidity, temperature cycling, salt exposure, mechanical fatigue, and electromagnetic interference. Without that level of validation, it is difficult to distinguish true deployment potential from well-executed proof-of-concept performance.

Extreme operational conditions. MXenes could function under a range of harsh conditions, but long-term stability under coupled thermal, mechanical, radiative, and corrosive stresses still requires major improvement. Elevated temperature, moisture, radiation, and repeated deformation can damage layered structures, alter termination chemistry, and raise interfacial resistance after MXenes are incorporated into chips, coatings, sensors, or structural hosts. Multilayer films may also delaminate under vibration or impact, especially when interfaces are weak or chemically unstable. Addressing these degradation pathways will require more than protective coatings alone; it will demand integrated control of terminations, interfaces, encapsulation, and mechanical architecture if MXenes are to serve as reliable materials in real military environments.

## 7. Summary

MXenes are a versatile family of two-dimensional transition-metal carbides and nitrides whose multifunctionality arises from selective-etching chemistry, tunable surface terminations, and layered architectures. Advances in synthesis, delamination, and post-treatment have greatly expanded the accessible compositional and structural space, establishing MXenes as an important platform for material systems that must couple electrical, mechanical, interfacial, and thermal functions within one architecture.

From a defense-oriented perspective, the significance of MXenes lies not in any single record metric, but in their ability to support multiple mission-relevant functions within limited mass and volume budgets. Their high conductivity and layered morphology favor lightweight electromagnetic shielding; their piezoresistive and electrochromic responses create opportunities for intelligent protection and adaptive regulation; and their promise in extreme-temperature energy storage and chemical sensing broadens their potential relevance to next-generation soldier systems, stealth-related platforms, and multifunctional energy architectures. At the same time, these opportunities are shaped by trade-offs among conductivity, environmental stability, emissivity, mechanical durability, and manufacturability.

Despite rapid progress, MXenes are not yet a mature deployment material for military systems. Their practical translation remains constrained by synthesis-related variability, termination heterogeneity, oxidation and interfacial degradation, and the limited availability of rigorous application-level validation. Future advances will therefore depend not only on further materials innovation, but also on scalable manufacturing, standardization of structure-property control, and integrated design across materials, devices, and systems. The most credible path forward is one that treats MXenes not as universally superior replacements, but as highly versatile building blocks whose value will be realized when their chemistry, architecture, and operating environment are engineered together.

## Figures and Tables

**Table 1:** Summary of Representative MXene Synthesis Methods.

Method	Mechanism	Advantages	Limitations
HF Etching	Strong fluorination, dissolution of A-site atoms	Fast, high purity	Safety hazards, poor termination control [58]
In-situ HF	Mild in-situ HF generation + cation intercalation	Safe, excellent delamination	Slow kinetics [59,60,61]
Bifluoride Etching	Bifluoride-derived fluoride release + cation intercalation	Simple, good layer expansion	Complex terminations [62,63]
Alkali Etching	A-layer dissolution in strong base	Fluorine-free	Harsh conditions [64,65]
Fluorinated Molten Salt	High-T molten fluoride etching	Access to nitride MXene	Difficult delamination [66,67]
Lewis Acid Molten Salt	Redox extraction + halide terminations	Termination-controlled	Produces multilayers [68,69,70]
Electrochemical Etching	Voltage-driven A-layer oxidation	Green, controllable	Low yield [71]
CVD	Direct deposition of MXene layers	High-quality films	Costly, not scalable [72,73]

## Data Availability

No new data were generated or analyzed in this study. Data sharing is not applicable to this article.

## DURC Statement

The authors acknowledge that MXene-based multifunctional materials may have potential dual-use implications because of their relevance to electromagnetic shielding, sensing, thermal regulation, and protective systems. However, the present work is limited to a scientific review of material synthesis, property regulation, and deployment challenges, and does not involve weapon design, operational guidance, or harmful applications. The intended applications are peaceful and civilian, including electromagnetic interference protection, flexible electronics, environmental sensing, energy storage, thermal management, and protective coatings. The authors are committed to ethical and responsible use of the reported findings, and all discussions are presented at the materials-science level, with no classified, weapon-specific, or misuse-enabling information included. Current research is limited to the field of MXene-based multifunctional materials and devices, which is beneficial for passive protection, electromagnetic shielding, thermal management, sensing, environmental monitoring, infrastructure resilience, and safety-related applications, and does not pose a threat to public health or national security. Authors acknowledge the dual use potential of the research involving MXene-based multifunctional materials and devices and confirm that all necessary precautions have been taken to prevent potential misuse. As an ethical responsibility, authors strictly adhere to relevant national and international laws about DURC. Authors advocate for responsible deployment, ethical considerations, regulatory compliance, and transparent reporting to mitigate misuse risks and foster beneficial outcomes.
